# Gender Differences and Effect of Air Pollution on Asthma in Children with and without Allergic Predisposition: Northeast Chinese Children Health Study

**DOI:** 10.1371/journal.pone.0022470

**Published:** 2011-07-19

**Authors:** Guang-Hui Dong, Tao Chen, Miao-Miao Liu, Da Wang, Ya-Nan Ma, Wan-Hui Ren, Yungling Leo Lee, Ya-Dong Zhao, Qin-Cheng He

**Affiliations:** 1 Department of Biostatistics and Epidemiology, School of Public Health, China Medical University, Shenyang, Liaoning Province, People's Republic of China; 2 Department of Occupational and Environmental Health, School of Public Health, China Medical University, Shenyang, Liaoning Province, People's Republic of China; 3 Department of Science and Technology, Educational Department of Liaoning Province, Shenyang, Liaoning Province, People's Republic of China; 4 Department of Ambient Air Pollution Monitor, Shenyang Environmental Monitoring Center, Shenyang, Liaoning Province, People's Republic of China; 5 Institute of Epidemiology and Preventive Medicine, College of Public Health, National Taiwan University, Taipei, Taiwan; 6 Institute of Respiratory Diseases, The First Affiliated Hospital of China Medical University, Shenyang, Liaoning Province, People's Republic of China; UCL Institute of Child Health, University College London, United Kingdom

## Abstract

**Background:**

Males and females exhibit different health responses to air pollution, but little is known about how exposure to air pollution affects juvenile respiratory health after analysis stratified by allergic predisposition. The aim of the present study was to assess the relationship between air pollutants and asthmatic symptoms in Chinese children selected from multiple sites in a heavily industrialized province of China, and investigate whether allergic predisposition modifies this relationship.

**Methodology/Principal Findings:**

30139 Chinese children aged 3-to-12 years were selected from 25 districts of seven cities in northeast China in 2009. Information on respiratory health was obtained using a standard questionnaire from the American Thoracic Society. Routine air-pollution monitoring data was used for particles with an aerodynamic diameter ≤10 µm (PM_10_), sulfur dioxide (SO_2_), nitrogen dioxides (NO_2_), ozone (O_3_) and carbon monoxide (CO). A two-stage regression approach was applied in data analyses. The effect estimates were presented as odds ratios (ORs) per interquartile changes for PM_10_, SO_2_, NO_2_, O_3_, and CO. The results showed that children with allergic predisposition were more susceptible to air pollutants than children without allergic predisposition. Amongst children without an allergic predisposition, air pollution effects on asthma were stronger in males compared to females; Current asthma prevalence was related to PM_10_ (ORs = 1.36 per 31 µg/m^3^; 95% CI, 1.08–1.72), SO_2_ (ORs = 1.38 per 21 µg/m^3^; 95%CI, 1.12–1.69) only among males. However, among children with allergic predisposition, more positively associations between air pollutants and respiratory symptoms and diseases were detected in females; An increased prevalence of doctor-diagnosed asthma was significantly associated with SO_2_ (ORs = 1.48 per 21 µg/m^3^; 95%CI, 1.21–1.80), NO_2_ (ORs = 1.26 per 10 µg/m^3^; 95%CI, 1.01–1.56), and current asthma with O_3_ (ORs = 1.55 per 23 µg/m^3^; 95%CI, 1.18–2.04) only among females.

**Conclusion/Significance:**

Ambient air pollutions were more evident in males without an allergic predisposition and more associations were detected in females with allergic predisposition.

## Introduction

Recent epidemiologic studies have revealed a wide variation in the increasing prevalence of asthma and allergies in many Asian countries, including mainland China [1], [2]. Comparative studies of the population of the same ethnic background living in different environments revealed important environmental risk factors for asthma, especially for ambient air pollution, which may play an important role in the development of asthma symptoms [3]–[6]. The contribution of ambient air pollution exposure to human respiratory disease seems to vary in different parts of the world, perhaps because there is a difference in spatial and temporal variability of air pollutants sources and composition between different regions [7], [8].

China is undergoing urbanization and westernization at an unprecedented rate, and the levels and patterns of outdoor and indoor air pollutants have altered dramatically. Ambient air quality has improved since the beginning of the 21^st^ century, and levels of indoor coal smoke pollution have decreased rapidly as people move to new houses with gas or electric power. But in many Chinese cities, the levels of thoracic particles (less than 10 µm in diameter [PM_10_]) and sulfur dioxide (SO_2_) are still higher than the air quality guidance value of the World Health Organization (WHO). The rapid increase in motor vehicle ownership and use has been accompanied by a concomitant increase in traffic-related air pollution, which poses an increasingly serious problem in urban areas of China. Consequently, urban air pollution in China has changed from the coal combustion type to a compound air pollution type due to the coexistence of coal smoke and motor vehicle emissions type. However, few studies have evaluated the health effects of this type of compound air pollution in China in the past decade, and to the best of our knowledge, no studies have considered the modified effects of not only gender difference but also the status of allergic predisposition when in the evaluating of the relationships between ambient air pollution and respiratory symptoms. Therefore, the effects of compound air pollution on human health require further study.

In the past, the outdoor levels of PM_10_ and SO_2_ in the province of Liaoning were among the highest in China. Current levels still exceed clean air guidelines of WHO, and, simultaneously, nitrogen oxides (NO_2_), carbon monoxide (CO), and ozone (O_3_) concentrations have significantly increased, albeit with substantial geographical variation. All the five pollutants were consistently low in one area near the coast of Yingkou and Dalian cities. This heavy but non-uniform air pollution in Liaoning province offers a valuable epidemiologic opportunity for the relationship between exposure and response to be assessed. In the present study, we investigated the associations between exposures to urban compound air pollution and respiratory health in school children, and also investigated whether allergic predisposition and gender modifies these relationships.

## Methods

### Ethics statement

The procedures followed were in accordance with ethical standards of the responsible committee on human experimentation of China Medical University. All human experiments performed herein were approved by the China Medical University Institutional Human Ethics Committee and were consistent with the principles outlined in NIH guidelines on the ethical conduct of human research. A written informed consent was obtained from the parent/guardian of each participant before data collection.

### Site Selection and Subject Recruitment

Liaoning province is located in northeast of China, and includes 14 cities. In order to maximize the inter- and intra-city gradients of the pollutants of interest and to minimize the correlation between district-specific ambient pollutants, in April 2009 we selected seven cities (Shenyang, Dalian, Anshan, Fushun, Benxi, Liaoyang, Yingkou) from Liaoning province based on the results of air pollution measurements between 2006 and 2008. There are five districts in Shenyang, four districts in Dalian and Fushun, and three districts in Anshan, Benxi, Liaoyang and Yingkou, respectively. An elementary school and two kindergartens within 1 km of air monitoring sites were randomly selected from each district. The resulting 25 elementary schools and 50 kindergartens were included with the 25 districts of the selected cities. Our entry criterion was “the subject should have lived at that place for at least 3 years”. The procedures followed were in accordance with ethical standards of the responsible committee on human experimentation of the China Medical University, and it complied with the principles outlined in the Helsinki Declaration. Endorsed consent forms were obtained from the parents or guardians of the children before their participation in the present study.

### Assessment of Ambient Air Pollution Exposure

Concentrations of PM_10_, SO_2_, NO_2_, O_3_, and CO in 2006–2008 were obtained from municipal air pollution monitoring stations. PM_10_, SO_2_, NO_2_, O_3_ and CO measurements at the municipal monitoring stations strictly followed the standard methods set by the State Environmental Protection Administration of China. Concentrations of each pollutant are measured continuously and reported hourly-PM_10_ by beta-attenuation, SO_2_ by ultraviolet fluorescence, NO_2_ by chemiluminescence, O_3_ by ultraviolet photometry, and CO by non-dispersive infrared spectrometry. A daily (24-hourly) averaged concentration was calculated when at least 13 valid hourly values were available with not more than 6 successive hourly values missing and an 8-hourly time-weighted average concentration was calculated when at least 6 valid hourly values were available. Exposure parameters in the present study were 3-year averages (2006–2008) and the yearly deviations from the 3-year average concentrations in each districts, calculated from the 24-hour PM_10_, SO_2_, NO_2_, CO, and 10:00 AM to 6:00 PM 8-hour O_3_.

### Questionnaire Survey

We included respiratory health related questions from the Epidemiologic Standardization Project Questionnaire of the American Thoracic Society (ATS-DLD-78-A) [9], which has been well validated in previous studies [3], [5], [6], [10], [11]. This questionnaire (ATS) has also been translated into Chinese for previous studies in other Chinese cities [3], [6], [11]. The questionnaire included detailed questions on date of birth, sex, birth weight, breast-feeding, number of years lived in each residence, types and characteristics of dwelling, method of cooking and heating, location of kitchen, types of home ventilation devices (if any), history and current status of children's respiratory illnesses and symptoms, parental education levels, smoking status of parents and other household members, and parental respiratory health histories. Local study staff informed students about the survey at the participating schools. After obtaining the written parental consent, parents were invited to a parents' night with the attendance of study staff where teachers explained the study and the conditions of consent. Parents who wished to complete the questionnaire at home returned it (via their child) to the teacher in an envelope. All questionnaire responses were recorded electronically in a database according to a standardized code and file structure.

### Definitions of Respiratory Symptoms and Illnesses

The following specific respiratory symptoms and illnesses were determined from questionnaire responses: ***a*** **)** ***Doctor diagnosed asthma:*** a yes answer to the question “Has a doctor ever diagnosed with asthma in this child?” ***b*** **)** ***Current asthma:*** under the condition that the child has been diagnosed asthma, a yes answer to the question “Has this child been in a paroxysm of asthma in last two years?” or a yes answer to the question “Does this child take medicine or treatment for asthma or asthmatic bronchitis?”. ***c*** **)** ***Current Wheeze:*** a yes answer to the question “Does this child's chest ever sound wheezy or whistling, including times when he or she had a cold?” and a yes answer to the question “Has this child had 2 or more such episodes in last 12 months?” ***d*** **)** ***Persistent cough:*** the answers to several cough-related questions which indicate that the study child has cough on most days (4 or more days per week) for at least 3 months a year either with or without cold during the last 12 months. ***e*** **)** ***Persistent phlegm:*** the answers to several phlegm-related questions which indicate that the study child has been seem congested or brought up phlegm, sputum, or mucus from the chest on most days (4 or more days per week) for at least 3 months a year either with or without cold during the last 12 months. ***f*** **)** ***Allergic rhinitis:*** a “yes” answer to the question “Has a doctor ever diagnosed allergic rhinitis in this child?”

***Family history of allergies*** was defined as any biological parent or grandparent in whom hay fever or allergies (including allergic dermatitis, allergic conjunctivitis, and eczema) had been diagnosed. ***Family history of asthma*** was defined as any biological parent or grandparents in whom asthma or bronchial asthma had been diagnosed. If subjects answer ‘yes’ to family history of allergy or family history of asthma, then they were defined as having an ***allergic predisposition***. ***Personal allergic history*** was defined by reports of the father or mother of the index child's ever having received a diagnosis of allergic constitution, allergic conjunctivitis or atopic eczema, and included any history of hay fever, allergies to food or medicine, inhaled dusts, pollen, molds, animal fur or dander, or skin allergies not including poison ivy and oak.

### Statistical Methods

The data was analyzed using SAS software (version 9.1; SAS Institute, Cary, N.C., USA). We studied the relationship between district-specific ambient levels of pollutants and prevalence rates of the following questionnaire-based morbidity end points: persistent cough, persistent phlegm, symptoms of asthma, current asthma, wheeze, and symptoms of wheeze. We estimated odds ratios in a two-stage hierarchical model using logistic and ecologic model analyses. The models assume two sources of variation: the variation among subjects in the first stage, part of which could be explained by the individual confounders, and the variation of air pollution between districts in the second stage, part of which could be explained by variables measured at the municipal level. In the analyses we assumed that 1) the outcome variable follows the Bernoulli distribution; 2) intercept terms are random at the municipal level; 3) all the explanatory variables are fixed effects. A logistic regression model was fitted in the first stage for the risk of asthma and asthma related symptoms as a function of site-specific intercepts, j, where αj = 1, …, 25, and personal covariates. The adjusted site-specific intercepts and prevalence rates are related by Pj = e^αj^/(1+e^αj^). In the second stage, these intercept terms representing the logit of the site-specific prevalence rates (Pj; j = 1, …, 25), adjusted for personal covariates, were regressed on each site-specific ambient pollutant level by using a linear “ecologic” regression, i.e., logit αj = α+Uj+βZj, where Uj denotes the random departure from the predicated prevalence αj on the logit scale for site j; Zj denotes the ambient pollution level for site j. Thus, β can be interpreted as the log odds ratio (per interquartile changes) for each pollutant, adjusted for personal characteristics. The results from the models are presented as odds ratios (ORs), along with their 95% confidence intervals (CIs). The goodness of fit was assessed with likelihood ratio tests (LR) to determine whether a variable contributed significantly to the model. First, we fitted a full model with a complete set of covariates. To elaborate sources of confounding, we fitted models with different combinations of covariates and compared the effect from models with and without the covariate of interest. If the covariate was with a probability value of less than 0.15 level for a given symptom, that covariate was retained and included in the final model. A two-tailed probability value of ≤0.05 was considered to be statistically significant.

## Results

### Characteristics of the Participants

There were a total of 35527 children in 25 elementary schools and 50 kindergartens, of which 31649 returned the questionnaire to give an overall response rate of 89.1%. The participation rates varied from 81.3% in Yingkou to 94.7% in Dalian, which did not correlated with either pollution levels or disease prevalence. In total, 1510 children (275 aged <3, 189 aged >13, 1046 residing in the current district for <3 years) were excluded from further analyses (exclusion rate of 4.8%). Among the 30139 children analyzed, the average age was 8.5 years (SD = 2.4 years) and 15203 (50.4%) were males. The gender ratios (male/female) varied from 48.1% in Yingkou to 50.5% in Benxi, and this variance was not significant (χ^2^ = 5.51, *p* = 0.48). For all children, the prevalence rates of doctor-diagnosed asthma, current asthma, current wheeze, allergic rhinitis, persistent cough, and persistent phlegm were 6.6%, 2.3%, 6.3%, 4.9%, 9.6%, and 4.6%, respectively. A total of 13.7% (4135/30139) of children were fitted for allergic predisposition, and the prevalence of allergic predisposition varied from 11.7% in Liaoyang to 17.4% in Benxi (χ^2^ = 70.8, *p*<0.01), whereas, this variance did not correlated with the pollutions levels both in males and in females. The characteristics of the participants with and without allergic predisposition were shown in Table 1. Compared with the children with allergic predisposition, children without allergic predisposition spent more time outdoors (17.6 hours/week vs. 8.4 hours/week; *p*<0.01).

**Table 1:** Characteristics of the children with (n = 4135) or without (n = 26004) allergic predisposition at 25 districts in northeast of China.

Characteristic	Children without Allergic Predisposition		Children with Allergic Predisposition	
	Male	Female	Male	Female
Asthma and asthma-related symptoms				
Persistent cough	1186(9.0)	1088(8.5)	311(15.0)	318(15.5)
Persistent phlegm	574(4.4)	478(3.7)*	164(7.9)	164(8.0)
Doctor-diagnosed asthma	822(6.3)	580(4.5)*	332(16.0)	254(12.4)*
Current asthma	263(2.0)	176(1.4)*	139(6.7)	102(5.0)*
Current wheeze	757(5.8)	579(4.5)*	293(14.1)	272(13.2)
Allergy rhinitis	651(5.0)	443(3.4)*	215(10.4)	172(8.4)*
Age (years)				
3∼6	3817(29.1)	3692(28.7)	599(28.8)	599(29.1)
7∼9	4879(37.2)	4901(38.1)	767(36.9)	771(37.5)
10∼12	4429(33.7)	4286(33.3)	712(34.3)	687(33.4)
Parental education<higher school	3637(28.0)	3472(27.0)	562(27.1)	546(26.5)
Breast feeding	11306(86.1)	11234(87.2)*	1840(88.6)	1876(91.2)*
Low birth weight	388(3.0)	429(3.3)	80(3.9)	63(3.1)
Obesity	816(6.2)	594(4.6)*	116(5.6)	68(3.3)*
Respiratory disease before 2 years old	2774(10.7)	2386(18.5)*	733(35.3)	611(29.7)*
Personal allergic history	1921(14.9)	2140(16.3)*	679(32.7)	641(31.2)
Numbers or room <3	7154(54.5)	6877(53.4)	1093(52.6)	1105(53.7)
House close to main road	2442(18.6)	2440(19.0)	440(21.2)	472(23.0)
House close to factory or chimney	2550(19.4)	2513(19.5)	508(24.5)	525(25.5)
Home decoration in recent 2 years	4416(33.7)	4346(33.7)	766(36.9)	709(34.5)
Home coal use	855(6.5)	837(6.5)	170(8.2)	165(8.0)
Ventilation device in kitchen	10878(82.9)	10706(83.1)	1688(81.2)	1703(82.8)
Air exchange in winter	7837(59.7)	7682(59.7)	1196(57.6)	1126(54.7)
Humidator use	1915(14.6)	1865(14.5)	305(14.7)	293(14.2)
Home carpet use	212(1.6)	157(1.2)*	36(1.7)	35(1.7)
House pets	1901(14.5)	2136(16.6)*	376(18.1)	390(19.0)
Passive smoking exposure				
Father	4845(36.9)	4705(36.5)	967(46.5)	956(46.5)
Mother	117(0.9)	91(0.7)	21(1.0)	32(1.6)
Anyone	6325(48.2)	6138(47.7)	1237(59.5)	1195(58.1)
Parents as responders	11690(89.1)	11652(90.5)*	1914(92.1)	1899(92.3)

Values are n (%).

*The difference between male and female is significant at the 0.05 level.

### Air Pollution Levels

Table 2 summarizes the distribution of the annual mean air pollutants concentrations in the 25 monitoring stations in the year 2006–2008. 72% to 100% of PM_10_ measurements, 32% to 96% of SO_2_, and 36% of NO_2_ exceeded the National Standard of China (State Environmental Protection Administration 1996) and WHO Air Quality Guidelines (WHO 2005) [12], [13]. There are wide variations between districts gradients for PM_10_ (79–171 µg/m^3^), SO_2_ (20–80 µg/m^3^), O_3_ (34–89 µg/m^3^) and CO (929–2911 µg/m^3^). The correlations between different pollutants between districts are shown in Table 3. The correlations among CO and NO_2_ (0.23), or O_3_ (0.26) tended to be relatively low across the 25 districts, with a higher correlation between PM_10_ and SO_2_ (0.78), and between PM_10_ and O_3_ (0.74). The district-specific ambient pollutant levels were stable over several years before and during the study.

**Table 2:** Distribution of 3-year average air pollutant concentrations (µg/m^3^) between 25 districts in seven cities of Liaoning Province 2006–2008.

	PM_10_	SO_2_	NO_2_	O_3_	CO
Mean ± SD	124.2±24.1	50.3±16.8	36.7±7.6	54.8±16.1	2045±578
Min	79	20	21	34	929
Max	171	80	51	89	2911
Interquartile Range*	31	21	10	23	1001
National standard†	100	60	40	160	4000
% of >NS	76	32	36	0	0
WHO guideline‡	20	20	40	100	–
% of >WHO	100	96	36	0	–

*Range from 25^th^ to 75^th^ percentile of district-specific concentrations.

† China national ambient air quality standard.

‡ WHO air quality guidelines, 2005.

Abbreviations: PM_10_, particles with aerodynamic diameter 10 µm or less; SO_2_, sulfur dioxide; NO_2_, nitrogen dioxide; CO, carbon monoxide; O_3_, ozone.

**Table 3:** Correlation coefficients of air pollutants across 25 districts.

	PM_10_	SO_2_	NO_2_	CO	O_3_
PM_10_	1	0.78*	0.70*	0.47*	0.74*
SO_2_		1	0.52*	0.32	0.67*
NO_2_			1	0.23	0.66*
CO				1	0.26
O_3_					1

Abbreviations: PM_10_, particles with aerodynamic diameter 10 µm or less; SO_2_, sulfur dioxide; NO_2_, nitrogen dioxide; CO, carbon monoxide; O_3_, ozone.

*p<0.05.

### Health Effects

Table 4 presents multivariate relationships between the risk of respiratory morbidity and potentially relevant personal or residential characteristics in the first-stage logistic regression. Respiratory diseases history at an early age, family history of atopic diseases, home decoration in recent the last 2 years, humidifier use at home, and passive tobacco smoke exposure at home significantly increased the OR for all six symptoms/illnesses. Significantly negative associations were observed between elder age, the gender, breast feeding, use of a ventilation device in the kitchen, air exchange in winter and some symptoms/illnesses.

**Table 4:** Adjusted odds ratios (OR) for personal and household covariates associated with respiratory morbidity.

Variable	Persistent cough	Persistent phlegm	Doctor-diagnosed asthma	Current asthma	Current wheeze	Allergic rhinitis
Age (ref: 3–6 years)						
7–9 years	0.67**	0.84**	1.04	0.69**	0.52**	2.34**
10–12 years	0.58**	0.84**	0.93	0.52**	0.39**	2.83**
Female (ref: male)	0.99	0.92	0.77**	0.76**	0.87**	0.73**
Parental education (ref: ≥junior high school)	1.18**	1.26**	1.34**	1.17*	1.01	0.92
Breast feeding (ref: not breast feeding)	0.83**	0.72**	0.78**	0.82*	0.95	0.68**
Low birth weight (ref: normal birth weight)	1.44**	1.49**	1.22*	1.03	1.16	1.30**
Obesity (ref: not obesity)	1.26**	1.37**	0.99	1.15	1.23**	1.28**
Respiratory disease history before 2 years old (ref: no)	2.72**	2.90**	8.68**	5.90**	4.97**	1.57**
Family history atopy (ref: no history)	1.46**	1.60**	1.49**	1.50**	1.78**	1.93**
Personal allergic history (ref: no history)	1.87**	2.05**	3.66**	5.43**	3.33**	7.39**
Numbers or room <3 (ref: ≥3 rooms)	1.13**	1.27**	1.12**	0.93	1.07	0.79**
House close to main road (ref: distance ≥20 m)	1.20**	1.14*	1.08	1.24**	1.11	0.98
House close to factory or chimney (ref: distance ≥100 m)	1.14**	1.06	1.11*	1.05	1.17**	0.98
Home decoration in recent 2 years (ref: not decoration in recent 2 yr)	1.28**	1.49**	1.19**	1.15*	1.16**	1.29**
Home coal use (ref: not coal use)	0.97	0.84*	1.07	0.88	1.12	0.85
Ventilation device in kitchen (ref: no device in kitchen)	0.78**	0.70**	0.91	0.91	0.99	1.02
Air exchange in winter (ref: no exchange in winter)	0.93*	0.91*	1.03	1.02	0.87**	1.10*
Humidator use (ref: no use)	1.26**	1.36**	1.30**	1.19*	1.15**	1.49**
Bedroom carpet use (ref: no carpet)	1.32*	1.02	1.34*	1.97**	1.30*	1.48**
House pets (ref: no pets in home)	1.48**	1.81**	1.27**	0.92	1.08	1.25**
Passive tobacco exposure at home (ref: no exposure)	1.33**	1.42**	1.23**	1.19**	1.40**	1.09*
Parents as responders (ref: others as responders)	0.86**	0.86*	1.01	1.07	1.10	0.72
Time spent outdoor (hour)	0.94	1.02	0.89*	0.75**	0.91	0.98

*p<0.15;

**p<0.05.

Items with asterisks are included in the final adjustment model for this measurement. These items are adjusted for each other; remaining variables are adjusted only for the footnoted items, as well as for districts.

For all participating children, we observed significant associations between ambient air pollutants and some symptoms/illness; when stratified by gender, there was little difference in these associations between males and females (data not shown). However, amongst children without an allergic predisposition, ambient air pollutants were associated with more respiratory symptoms and diseases in males than in females (Table 5). For instance, in the single pollutant model analysis, positive associations were identified for current asthma with interquartile range of PM_10_ (OR = 1.36, 95%CI: 1.08–1.72), SO_2_ (OR = 1.38, 95%CI: 1.12–1.69), and doctor diagnosed asthma with NO_2_ (OR = 1.19, 95%CI: 1.06–1.34) only in males, but not in females. Among children with an allergic predisposition, females were more susceptible to ambient air pollutants than males (Table 6). In females, an increased prevalence of doctor diagnosed asthma was significantly associated with an interquartile range of SO_2_ (OR = 1.48; 95%CI: 1.21–1.80), NO_2_ (OR = 1.26; 95%CI: 1.01–1.56), and current asthma with O_3_ (OR = 1.55; 95%CI: 1.18–2.04), but in males, these relationships were not identified. Even for the PM_10_, the adjusted OR in females were higher than that in males.

**Table 5:** Adjusted OR and 95% CIs of respiratory diseases with respect to ambient air pollutants (2006–2008) among children without allergic predisposition (n = 26004)†.

Pollutant		PM_10_	SO_2_	NO_2_	O_3_	CO
***Single pollutant model*** ‡						
Males	Persistent cough	**1.12(1.02–1.23)**	1.03(0.95–1.12)	**1.28(1.16–1.41)**	**1.14(1.04–1.26)**	**1.20(1.06–1.35)**
	Persistent phlegm	1.11(0.96–1.27)	1.02(0.91–1.14)	**1.16(1.02–1.33)**	1.08(0.95–1.24)	**1.24(1.04–1.48)**
	Doctor-diagnosed asthma	**1.27(1.12–1.45)**	**1.19(1.06–1.33)**	**1.19(1.06–1.34)**	**1.21(1.08–1.36)**	1.11(0.94–1.31)
	Current asthma	**1.36(1.08–1.72)**	**1.38(1.12–1.69)**	1.08(0.89–1.30)	1.09(0.90–1.33)	1.21(0.90–1.63)
	Current wheeze	1.11(0.98–1.26)	**1.10(1.00–1.24)**	1.00(0.89–1.12)	1.06(0.93–1.19)	**1.15(1.00–1.30)**
	Allergy rhinitis	**1.49(1.29–1.73)**	**1.42(1.24–1.62)**	**1.56(1.35–1.81)**	**1.49(1.30–1.72)**	1.11(0.71–1.72)
Females	Persistent cough	**1.12(1.00–1.25)**	1.03(0.94–1.12)	**1.21(1.09–1.33)**	1.06(0.96–1.17)	**1.15(1.02–1.29)**
	Persistent phlegm	1.01(0.89–1.15)	1.01(0.89–1.15)	1.13(0.98–1.30)	1.06(0.92–1.23)	1.15(0.98–1.34)
	Doctor-diagnosed asthma	**1.18(1.05–1.33)**	**1.15(1.04–1.28)**	1.14(0.99–1.30)	**1.17(1.02–1.33)**	0.96(0.84–1.10)
	Current asthma	1.16(0.95–1.41)	1.06(0.89–1.25)	1.11(0.88–1.40)	1.15(0.91–1.45)	0.94(0.74–1.18)
	Current wheeze	1.05(0.94–1.18)	1.02(0.92–1.13)	0.96(0.85–1.10)	1.01(0.88–1.16)	1.13(0.95–1.33)
	Allergy rhinitis	**1.39(1.22–1.57)**	**1.23(1.10–1.37)**	**1.51(1.33–1.71)**	**1.30(1.15–1.46)**	1.02(0.79–1.30)
***Multi-pollutant (five pollutants) model*** ‡						
Males	Persistent cough	0.96(0.80–1.15)	0.86(0.75–0.98)	**1.36(1.19–1.56)**	1.06(0.90–1.24)	**1.16(1.02–1.32)**
	Persistent phlegm	0.74(0.58–0.96)	0.99(0.82–1.19)	**1.26(1.05–1.51)**	1.15(0.91–1.44)	**1.23(1.03–1.47)**
	Doctor-diagnosed asthma	**1.36(1.06–1.76)**	1.05(0.87–1.27)	0.97(0.80–1.16)	0.91(0.72–1.15)	0.95(0.79–1.15)
	Current asthma	**1.54(1.09–2.19)**	1.46(0.94–2.27)	0.88(0.63–1.23)	0.64(0.40–1.00)	0.95(0.68–1.33)
	Current wheeze	1.03(0.82–1.28)	0.94(0.79–1.11)	0.92(0.78–1.09)	1.13(0.91–1.40)	1.14(0.97–1.34)
	Allergy rhinitis	**1.46(1.13–1.89)**	1.01(0.85–1.20)	**1.36(1.15–1.61)**	0.86(0.71–1.04)	0.97(0.80–1.19)
Females	Persistent cough	1.11(0.92–1.33)	0.88(0.76–1.01)	**1.30(1.13–1.50)**	0.88(0.74–1.05)	**1.18(1.03–1.35)**
	Persistent phlegm	1.08(0.83–1.42)	0.86(0.70–1.06)	1.15(0.94–1.42)	0.98(0.76–1.27)	**1.24(1.02–1.52)**
	Doctor-diagnosed asthma	**1.38(1.01–1.94)**	0.93(0.71–1.22)	0.97(0.74–1.26)	0.96(0.69–1.34)	0.83(0.63–1.09)
	Current asthma	1.08(0.87–1.36)	1.05(0.89–1.22)	1.08(0.93–1.27)	1.07(0.88–1.30)	0.87(0.75–1.02)
	Current wheeze	1.19(0.94–1.52)	1.17(0.96–1.43)	0.85(0.71–1.03)	0.84(0.65–1.09)	1.04(0.86–1.26)
	Allergy rhinitis	1.31(0.96–1.79)	1.16(0.94–1.43)	**1.31(1.07–1.59)**	0.99(0.78–1.24)	0.98(0.77–1.23)

† Models were adjusted for the variables with asterisks in table 4.

‡ OR were scaled to the interquartile range for each pollutant (31 µg/m^3^ for PM_10_, 21 µg/m^3^ for SO_2_, 10 µg/m^3^ for NO_2_, 1001 µg/m^3^ for CO, and 23 µg/m^3^ for O_3_).

**Table 6:** Adjusted OR and 95% CIs of respiratory diseases with respect to ambient air pollutants (2006–2008) among children with allergic predisposition (n = 4135)†.

Pollutant		PM_10_	SO_2_	NO_2_	O_3_	CO
***Single pollutant model*** ‡						
Males	Persistent cough	**1.31(1.08–1.59)**	**1.28(1.07–1.52)**	**1.26(1.04–1.53)**	**1.32(1.09–1.59)**	1.10(0.86–1.40)
	Persistent phlegm	**1.40(1.08–1.82)**	**1.30(1.04–1.63)**	1.24(0.96–1.61)	**1.37(1.07–1.75)**	1.30(0.95–1.79)
	Doctor-diagnosed asthma	**1.31(1.08–1.60)**	1.18(0.99–1.41)	1.23(0.99–1.52)	**1.30(1.05–1.61)**	0.98(0.77–1.25)
	Current asthma	**1.52(1.14–2.05)**	**1.37(1.05–1.77)**	**1.38(1.04–1.82)**	1.10(0.79–1.51)	0.95(0.67–1.35)
	Current wheeze	1.13(0.92–1.38)	1.03(0.86–1.24)	0.99(0.81–1.20)	0.99(0.81–1.23)	1.01(0.78–1.30)
	Allergy rhinitis	**1.47(1.16–1.85)**	**1.47(1.19–1.82)**	**1.47(1.17–1.83)**	**1.52(1.22–1.89)**	0.87(0.66–1.14)
Females	Persistent cough	**1.37(1.13–1.66)**	**1.28(1.08–1.53)**	**1.40(1.15–1.70)**	**1.43(1.19–1.75)**	1.16(0.91–1.47)
	Persistent phlegm	**1.44(1.12–1.87)**	**1.34(1.06–1.69)**	**1.35(1.05–1.74)**	**1.64(1.27–2.11)**	**1.35(1.01–1.82)**
	Doctor-diagnosed asthma	**1.46(1.17–1.83)**	**1.48(1.21–1.80)**	**1.26(1.01–1.56)**	**1.41(1.16–1.72)**	0.98(0.75–1.27)
	Current asthma	**1.59(1.15–2.22)**	**1.61(1.20–2.15)**	**1.44(1.04–1.98)**	**1.55(1.18–2.04)**	1.16(0.78–1.73)
	Current wheeze	1.15(0.93–1.42)	1.07(0.89–1.29)	1.08(0.87–1.33)	**1.25(1.02–1.53)**	1.07(0.83–1.39)
	Allergy rhinitis	**1.74(1.34–2.26)**	**1.55(1.23–1.96)**	**1.73(1.35–2.21)**	**1.68(1.33–2.11)**	1.23(0.87–1.74)
***Multi-pollutant (five pollutants) model*** ‡						
Males	Persistent cough	0.92(0.64–1.34)	1.07(0.81–1.40)	1.18(0.90–1.56)	1.31(0.93–1.83)	0.99(0.75–1.31)
	Persistent phlegm	1.08(0.64–1.82)	1.05(0.73–1.52)	1.14(0.79–1.65)	1.11(0.71–1.72)	1.14(0.78–1.65)
	Doctor-diagnosed asthma	1.27(0.87–1.87)	0.88(0.66–1.16)	0.91(0.69–1.21)	**1.42(1.01–1.99)**	0.85(0.64–1.12)
	Current asthma	1.40(0.80–2.44)	1.05(0.71–1.54)	0.94(0.63–1.40)	1.28(0.79–2.07)	0.75(0.50–1.11)
	Current wheeze	1.39(0.93–2.08)	1.02(0.75–1.39)	1.07(0.79–1.46)	0.73(0.50–1.06)	0.95(0.70–1.29)
	Allergy rhinitis	1.17(0.73–1.86)	1.28(0.93–1.76)	1.16(0.84–1.58)	1.11(0.77–1.58)	070(0.51–0.96)
Females	Persistent cough	1.24(0.84–1.81)	1.08(0.82–1.42)	1.03(0.78–1.36)	1.04(0.74–1.46)	0.97(0.73–1.28)
	Persistent phlegm	0.96(0.58–1.61)	0.97(0.67–1.38)	0.79(0.54–1.14)	**1.98(1.25–3.13)**	1.22(0.84–1.77)
	Doctor-diagnosed asthma	**1.54(1.01–2.39)**	**1.45(1.06–1.98)**	0.97(0.72–1.33)	0.78(0.53–1.13)	0.69(0.51–0.95)
	Current asthma	**2.04(1.24–3.35)**	**1.91(1.00–3.67)**	1.34(0.84–2.15)	0.33(0.19–0.60)	0.72(0.45–1.16)
	Current wheeze	1.13(0.77–1.67)	0.82(0.62–1.09)	0.73(0.54–0.97)	**1.66(1.16–2.38)**	0.97(0.73–1.30)
	Allergy rhinitis	**1.84(1.06–3.19)**	1.15(0.81–1.63)	1.19(0.84–1.69)	0.99(0.67–1.45)	0.50(0.35–0.71)

† Models were adjusted for the variables with asterisks in table 4.

‡ OR were scaled to the interquartile range for each pollutant (31 µg/m^3^ for PM_10_, 21 µg/m^3^ for SO_2_, 10 µg/m^3^ for NO_2_, 1001 µg/m^3^ for CO, and 23 µg/m^3^ for O_3_).

## Discussion

In present study, the average levels of PM_10_ and SO_2_ were around 6 and 2.5 times higher than WHO recommended limit respectively, substantially extending the upper end of the pollution ranges of previous epidemiological studies conducted in Europe [14] and North America [5], [10], and Taiwan [3]. These wide gradients and ‘natural’ experiment offered us the opportunity to study the effect of exposure to compound air pollution on the prevalence of respiratory symptoms among children within the high pollution range. Also, the ecologic exposure assessment had many advantages in our study. Compared to European and American children, the residential mobility of Chinese children is low and the density of elementary schools in China is very high. Monitoring stations located near the schools were also likely to be near the students' homes and thus provided good indicators for both school and home exposure.

A number of studies have evaluated the effects of ambient air pollution on children's respiratory health in the past two decades, however, the inconsistent results from these studies do not provide a clear overall picture of health damage [3], [5], [6], [10], [11], [14], [15]. In the early Harvard trial performed in six cities, respiratory morbidity outcomes in children were positively associated with ambient levels of PM_10_, SO_2_ and O_3_ [10]. A recent population-based nested case-control study also found that the prevalence of respiratory symptoms was associated with increased concentration of NO, NO_2_ and CO [15]. Even at relatively low ambient concentrations, ambient air pollution also independently contributed to the burden of emergency department visits for pediatric asthma [16]. On the other hand, a study of 12 communities of Southern California did not show any association between ambient air pollutants (PM_10_, O_3_, and NO_2_) and respiratory illness in children except for acid with wheezing (OR = 1.26 per 1.7 ppb increase in acid) [5]. A study of four Chinese cities, which was conducted between 1993 and 1996, also showed a significantly increased prevalence of persistent phlegm (OR = 3.21, 95% CI:1.55–6.67) for an interquartile increment of PM_10_ (87 µg/m^3^) among children, but did not reveal a relationship between ambient NO_2_ or SO_2_ levels and the prevalence of respiratory symptoms [6]. Also, a more recent one-city study did not show any significant relationship between the outdoor levels of NO_2_, SO_2_, and O_3_ and increased of wheeze and asthma in children from Taiyuan city of China [11]. By selecting 25 districts from 7 cities, we have demonstrated the characteristics of the overall harmful effects which could not have been evaluated in one-city studies.

There is growing epidemiological evidence of the differing associations between air pollution and respiratory health for males and females, but the literature is far from consistent [17], [18]. The gender analyses are more common in occupational epidemiology than in environmental health, because persistent job stratification by gender has produced marked differences in occupational exposures to chemical agents, ergonomic demands, injury, and psychosocial stressors [17]. Compared with the studies among adults, disentangling gender effects in air pollution–health associations among children may be more complicated, because lung function growth rates (critical periods for pollution effects) differ by sex [19]. In our study, the vulnerability towards exposure to ambient air pollutants appeared to differ between males and females after stratified analyses by the status of allergic predisposition (Table 5 and Table 6). Consistent with our findings in children without an allergic predisposition, several studies indicated that the airways of males and females respond differently to exposure to air pollutants [5], [20]–[22]. This is plausible as there are differences between male and female airways from early in fetal lung development and throughout life [23], [24], for example female lungs mature earlier with regard to surfactant production. Throughout life women have smaller lungs than men, but their lung architecture is more advantageous with a greater airway diameter in relation to the volume of the lung parenchyma. Thus, in childhood, airway hyper-responsiveness and asthma is more common among boys than girls. As shown in the present study, among the children without an allergic predisposition, the stronger association between ambient air pollutants exposure and respiratory symptoms and diseases in males (Table 5), could be related to males having less mature lungs and relatively narrower airways during childhood. These factors are believed to contribute to the generally higher rates of pulmonary morbidity in boys than in girls, and could possibly also explain a higher susceptibility for damage by exposure to air pollutants during this age window.

Because there is little information in the literature regarding the gender-specific effects of ambient air pollution on respiratory symptoms/illness among children with an allergic predisposition, it is difficult to compare the results of our current studies with those of other investigations. First, children with an allergic predisposition spend more time at home compared with children without an allergic predisposition, so their exposure to air pollutants may be different. In the six Cities study, Neas et al. [25] reported stronger associations between home indoor NO_2_ and respiratory symptoms among girls (OR = 1.7, 95%CI: 1.3–2.2) than among boys (OR = 1.2, 95%CI: 0.9–1.5) 7–11 years of age. Results from an Italian study showed there was no association between time spent in the kitchen and lung function level in boys [26], but a reduction in lung function was detected in girls which was statistically significant for FEF_75_ (sometimes –10.3%, often –11.1%), and after stratifying boys and girls into four groups on the basis of the IgE serum level (below and above the median value of IgE), the reduction in lung function was significant in girls with a high IgE value whereas no significant deleterious effects were evident in girls with a low IgE value or in boys with either a low or high IgE. So, our results (Table 5 and Table 6), in combination with those from Corbo and colleagues, suggest that when exposed to indoor air pollutants, females with an allergic predisposition may be more susceptible than females without an allergic predisposition or males either with or without an allergic predisposition. Secondly, compared with allergic asthmatics, non-allergic asthmatics have a higher sensibility of nasal and bronchial epithelia to non-allergenic stimuli like air pollutants, strong smells, cold air, wind or respiratory viruses [27]. As previously reported [28], clinical history of atopy was significantly more frequent in allergic asthmatics, and hay fever was the factor that most lowered the risk of displaying non-allergic asthma. Among the asthmatics with allergic predisposition of this study, we found 40.1% of personal allergic history and 17.3% of history hay fever in females, which was significantly lower than that in males (59.9% and 35.1%, respectively). Also, Romanet-Manent et al. [27] reported that the female sex is associated with an increased risk of non-allergic asthma as compared to allergic asthma. Therefore, we may hypothesize that, amongst children with an allergic predisposition, females may be more likely to display a non-allergic type of asthma comparing with males, and therefore may be more sensitive to air pollutants than males. The difference of health response to air pollution between males and females remains unclear and needs to be explored further. Careful consideration of gender effects and exploration of nascent methods for quantitative gender analysis may help to elucidate sources of difference.

The strong correlations between PM_10_ and SO_2_ (r = 0.78), PM_10_ and NO_2_ (r = 0.70), PM_10_ and O_3_ (r = 0.74), SO_2_ and O_3_ (r = 0.67), NO_2_ and O_3_ (r = 0.66) make it difficult to distinguish the effects of individual air pollutant. The increased ORs for PM_10_ and decreased ORs for SO_2_, NO_2_ and O_3_ in multi-pollutant models may reflect the dominated influence of particulate material pollution in the ‘coal smoke’ style air pollution in the seven cities of northeast China [29]. Rather than being itself responsible for the increased risks of respiratory symptoms, PM_10_ maybe operating as the best surrogates of the mixed air pollutants in this context. The decreased ORs for SO_2_, NO_2_ and O_3_ may be caused by the limited effectiveness of multi-pollutant regression models in controlling for confounding by co-pollutants [30].

Our study has some limitations. This was a study of respiratory prevalence in children between 3 and 12 years of age, not of respiratory symptom incidence; for example, some of the factors that we studied might have affected respiratory symptoms prevalence through effects on diseases duration rather than disease incidence. Also, this is a cross-sectional study and a temporal relationship between exposure and outcome could not be established.

In conclusion, our data confirm that ambient compound air pollution was associated with respiratory symptoms and diseases in young children. Among children without an allergic predisposition, males might be more susceptible to ambient air pollution than females; whereas among children with an allergic predisposition, more associations were detected in females in this group. These observations further support the results of published studies that ambient air pollution may cause respiratory symptoms and allergic diseases in children. Additionally, researchers should track these responses across multiple generations to identify potential long-term risks of exposures and the influence of gender on that risk.
